# Data for Sochi 2014 Olympics discussion on social media

**DOI:** 10.1016/j.dib.2017.06.040

**Published:** 2017-06-27

**Authors:** Andrei P. Kirilenko

**Affiliations:** The Department of Tourism, Recreation and Sport Management, University of Florida, P.O. Box 118208, Gainesville, FL 32611-8208, United States

**Keywords:** Mega sports event, Olympic Games, Classification, Social networks, Twitter

## Abstract

Presented data is related to the research article “Sochi 2014 Olympics on Twitter: Perspectives of Hosts and Guests” [2].  The data were collected through regular API Twitter search for five months windowing 2014 Sochi Olympic Games and further used for cluster analysis and analysis of the sentiment on the Games. The main dataset contains 616 thousand tweets, rigorously cleaned and filtered to remove irrelevant content. To comply with the Twitter API user agreement, the dataset presented in this article includes only generalized daily data with all information contained in individual tweets removed. The proposed use of the dataset is academic research of changing discussion on the topics related to Mega-events in conjunction with political events.

**Specifications Table**

**Table:** 

Subject area	*Social sciences*
More specific subject area	*Social media*
Type of data	*Database*
How data was acquired	*API search*
Data format	*processed*
Experimental factors	
Experimental features	
Data source location	https://dataverse.harvard.edu/privateurl.xhtml?token=92d98b28-4583-4d3c-a707-e90c71141163
Data accessibility	*Online*

**Value of the data**

• Hashtags (keywords) are an emerging linguistic norm; as such are widely used in social media research and data mining to discern changes in main topics and sentiment of public discourse.
• Database represents 613 thousand tweets, filtered, quality-evaluated, stripped of identifying content and generalized on a daily basis to comply with Twitter API data use agreement.
• Additional datasets represent the hashtags contained in English and Russian messages that represent the majority of tweets in collected data.

## 1 Data

Mega-events such as the Olympic Games create new opportunities for communities and businesses and stimulate economic growth [4]. At the same time, the increased international country visibility promotes discussion of the issues only laterally related to main event such as human rights, politics, environment, etc. An emerging method for public opinion mining surrounding international mega-events employs data from online social media such as blogs and photo sharing platforms. After the data is collected, data mining methods are used to discern main topics of public interest, geographical patterns, changes in positive and negative emotions, and similar derived variables. This way, social media analytics has been successfully applied in diverse areas such as disaster management, election polls, and in formulating relevant social policies [1,3].

**Fig. 1:**
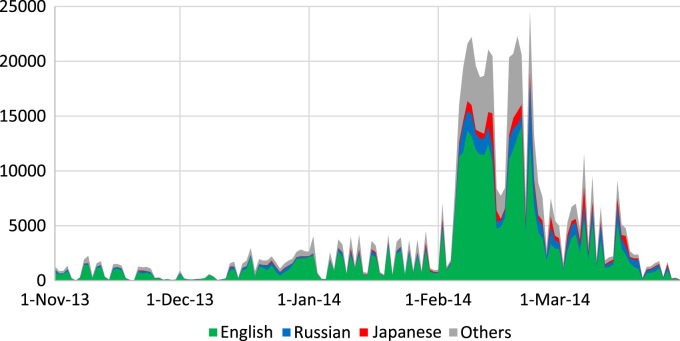
Daily number of collected tweets for different languages. Primary peaks in data collection correspond to the beginning and the end of the Olympic Games (February 7–23, 2014) and the secondary peak corresponds to Paralympics (March 7–16, 2014).

The published dataset (Supplementary Database 1; see metadata in Table 1) reflects the online discussion of 2014 Sochi Winter Olympic Games (February 7–23, 2014) and Winter Paralympic Games (March 7–16, 2014) on Twitter microblogging platform. The data shows the most frequent hashtags (keywords) contained in tweets published prior and immediately after the Olympic Games, allowing tracking changes in public discourse prior, during, and after the mega-event. To comply with the Twitter Terms of Service, raw data are not shared; the derived product contains frequency of the most frequent hash tags on a daily bases, stripped of any geographical or personal identification. The dataset was used in related research article [2], which contrasted main themes of discussion in Russian and English sectors of Twitter and compared pre- and post-Games sentiment expressed by the Olympic Games hosts and guests. Fig. 1 depicts daily number of collected tweets in different languages.

**Table 1:** Database tables.

**Table name**	**Content**	**Columns**
dailyhashtags	Daily number of collected hashtags	Lang: Language code (IANA language subtag registery)
		yearSochi: year standardized to GMT-3
		monthSochi: month standardized to GMT-3
		daySochi: day standardized to GMT-3
		nday: Day number (d1=November 1, 2013 GMT-3)
		Count: number of hashtag usage
dailyHashtagsEn	Daily number of the most frequent hashtags (English language)	d1.d150: Day number (d1=November 1, 2013)
dailyHashtagsRu	Daily number of the most frequent hashtags (Russian language)	d1.d150: (d1=November 1, 2013)
hashEn	Overall number of collected tweets (English language)	hashtag: hashtag
		Ntweets: number of tweets with this hashtag
		Freq. 1: hashtag frequency (including the most frequent)
		Freq. 2: hashtag frequency (excluding the most frequent)
		Rank: rank
hashRu	Overall number of collected tweets (Russian language)	hashtag: hashtag
		Ntweets: number of tweets with this hashtag
		Freq. 1: hashtag frequency (including the most frequent)
		Freq. 2: hashtag frequency (excluding the most frequent)
		Rank: rank
hashtags	List of hashtags in collected tweets (found at least 14 times in collected data), together with a common synonym where applicable	hashtag: hashtag
		Synonym: common synonym
		Count: total number of hashtags in the dataset

## 2 Experimental design, materials and methods

Raw data were collected through Twitter REST API search with adaptive frequency (from six times daily and up to one time every three minutes during the Games) using the key words *sochi*, *olympics*, *paralympics* and their translations into Russian for one year, starting November 1st, 2013, resulting in 7.8 million tweets. For quality control, a stratified random sampling was applied to collected data and the selected sample of 600 tweets was manually classified to two classes of those related and unrelated to Sochi Olympics. The manually classified sample was used to extract classification rules, which were applied to filter collected data based on (1) month of data collection and (2) hash tags with the goal of minimizing percentage of tweets unrelated to the Games, which resulted in the retained subset of 616,333 tweets spanning from November 1st, 2013 to March 31st, 2014.

For quality assessment, an independent sample of the retained data was evaluated; it was concluded that for each of the five months from November, 2013 to March, 2014 in data collection the final dataset contained at least 85% of relevant tweets (Table 2). Note that this evaluation was applied only to those tweets published in two most common languages in the dataset, English and Russian; in total, these tweets represent 439,106 tweets out of 616,333. Accordingly, the provided data contain language attribute.

**Table 2:** Percentage of relevant tweets in Russian and in English in the database.

Month	Russian	English
11/2013	95	100
12/2013	100	85
1/2014	90	95
2/2014	95	100
3/2014	85	100

## References

[1] Grubmüller V., Krieger B., Gotsch K. (2013). Social media analytics for future oriented policy making. Eur. J. Futur. Res..

[2] A. Kirilenko, S. Stepchenkova, Sochi-2014 Olympics on Twitter: Perspectives of hosts and guests, Tour. Manag., 63, 2017, 54-65.

[3] Margo M.J. (2012). A review of social media use in e-govenrment. Adm. Sci..

[4] P. Matos, Hosting mega sports events – a brief assessment of their multidimensional impacts, in: Proceedings of the Copenhagen Conference on the Economic and Social Impact of Hosting Mega Sports Events, 1, 2006.

